# Safety and Efficacy of Cerebrolysin for Neurorecovery After Acute Ischemic Stroke: A Systematic Review and Meta-Analysis of 14 Randomized Controlled Trials

**DOI:** 10.7759/cureus.91054

**Published:** 2025-08-26

**Authors:** Parag N Patel, Devang Mangal, Krunal Patel

**Affiliations:** 1 Medicine, Gujarat Medical Education & Research Society (GMERS) Medical College and Hospital, Patan, IND; 2 Medicine, Gujarat Medical Education & Research Society (GMERS) Medical College and Hospital, Ahmedabad, IND

**Keywords:** acute ischemic stroke, cerebrolysin, meta-analysis, neurorecovery, nihss

## Abstract

Cerebrolysin, a neurotrophic compound, has been investigated as a neurorestorative therapy in acute ischemic stroke, although findings are inconsistent due to limitations in study inclusion and outcome reporting. This systematic review and meta-analysis evaluated the efficacy and safety of Cerebrolysin in improving neurological and functional outcomes following acute ischemic stroke. Fourteen randomized controlled trials (N = 2,884 patients) comparing Cerebrolysin to placebo were included. The primary outcome was change in the National Institutes of Health Stroke Scale (NIHSS) score from baseline to follow-up, while secondary outcomes included functional independence (modified Rankin Scale (mRS) 0-2), serious adverse events (SAEs), mortality, and hemorrhagic transformation. Risk of bias was assessed using the revised Cochrane Risk of Bias (RoB 2.0) tool, and certainty of evidence was evaluated using the Grading of Recommendations, Assessment, Development, and Evaluation (GRADE) framework. Pooled analysis showed that Cerebrolysin significantly improved neurological recovery (mean difference in NIHSS change: +1.39; 95% confidence interval (CI): 0.53-2.25; p = 0.020). Functional independence showed a non-significant trend in favor of Cerebrolysin (risk ratio (RR) = 1.31; 95% CI: 0.90-1.91; p > 0.05). No significant differences were observed in SAEs (RR = 1.08; 95% CI: 0.84-1.40), mortality (RR = 0.86; 95% CI: 0.68-1.09), or hemorrhagic transformation (RR = 0.55; 95% CI: 0.32-0.92). These findings suggest that Cerebrolysin significantly enhances early neurological recovery after ischemic stroke, with a comparable safety profile. Further high-quality trials are warranted to confirm its impact on long-term functional outcomes.

## Introduction and background

Stroke remains a leading cause of mortality and long-term disability worldwide, with ischemic strokes comprising approximately 85% of all cases. The devastating impact of stroke is further underscored by the occurrence of sudden and unexpected deaths, often from non-traumatic intracerebral or subarachnoid hemorrhage, which claims lives without warning and contributes significantly to overall stroke mortality [1]. Although acute interventions-such as intravenous thrombolysis and endovascular thrombectomy-have improved early outcomes, a significant proportion of patients continue to experience incomplete neurological recovery [2]. This has driven growing interest in adjunctive neurorestorative therapies targeting the subacute phase of stroke.

Cerebrolysin is a multimodal neuropeptide preparation derived from porcine brain proteins, with demonstrated neuroprotective and neurorestorative effects in preclinical models [3]. Its proposed mechanisms include anti-apoptotic effects, attenuation of oxidative stress, promotion of neurogenesis, and enhancement of synaptic plasticity [3,4]. Based on these actions, it has been evaluated in clinical trials for post-stroke recovery, typically initiated within the first 48 hours after symptom onset.

Over the past two decades, several meta-analyses have explored the effects of Cerebrolysin in acute ischemic stroke [5,6]. While several reported improvements in outcomes (such as National Institutes of Health Stroke Scale (NIHSS) reduction and higher rates of functional independence (modified Rankin Scale (mRS) 0-2)), their conclusions have been limited by key methodological flaws. Many included studies with varying designs or mixed stroke subtypes [7-9], lacked consistent safety data, or failed to stratify results by follow-up duration [9]. In addition, older trials often showed inconsistencies in risk of bias assessments and signs of publication bias [10-13].

Since the most recent major meta-analysis [6], several new randomized controlled trials (RCTs), including those by Khasanova and Kalinin [14] and Homberg et al. [15], have been published but not yet incorporated into pooled analyses. Moreover, continued reliance on older trials-many with unclear or high risk of bias-has raised concerns about the overall certainty of the evidence base [16,17]. A 2023 Cochrane review by Ziganshina et al. also assessed Cerebrolysin, but its inclusion of non-randomized and outdated studies further limits generalizability [18].

This meta-analysis was explicitly designed to address the shortcomings of prior reviews by implementing a more rigorous and targeted methodology. Only randomized, placebo-controlled, parallel-group trials were included to reduce bias and enhance comparability across studies. Notably, we incorporated several recently published trials previously absent from earlier analyses, ensuring a more current and complete synthesis.

We stratified treatment effects by outcome type-including changes in NIHSS scores and rates of functional independence (mRS 0-2), as well as by follow-up duration, to better understand the magnitude and time course of Cerebrolysin’s effects. In addition to efficacy, we comprehensively assessed safety outcomes: serious adverse events (SAEs), mortality, and hemorrhagic transformation (HT). Our approach adheres to current best practices in systematic review methodology, including revised Cochrane Risk of Bias (RoB 2.0) for bias assessment, Grading of Recommendations, Assessment, Development, and Evaluation (GRADE) for evidence certainty, and Preferred Reporting Items for Systematic reviews and Meta-Analyses (PRISMA) 2020 for reporting transparency. Taken together, this study provides the most robust and up-to-date evaluation of Cerebrolysin’s therapeutic potential in early post-ischemic stroke recovery.

## Review

Materials and methods

Study Selection and Information Sources

The research question was framed using the PICO approach, focusing on adult patients with acute ischemic stroke (population), treated with Cerebrolysin infusion (intervention), compared to placebo or saline (comparator), and assessed for both efficacy and safety outcomes. The study protocol was developed prior to data extraction, and existing registrations were screened in PROSPERO to prevent duplication of prior reviews. A comprehensive systematic search was conducted across PubMed, Embase, and the Cochrane Central Register of Controlled Trials (CENTRAL) from inception until July 10, 2025. The protocol for this systematic review was registered with PROSPERO (CRD4201108156). The primary search terms included “Cerebrolysin” AND “stroke” AND “randomized controlled trial,” without applying filters. Additional records were identified through manual screening of relevant systematic reviews, trial registries (ClinicalTrials.gov and ISRCTN), and citation chaining. All search outputs were de-duplicated using DOIs and titles and exported into structured spreadsheets for review.

The following 14 RCTs were included: Lang et al. (2012) [7], Xue et al. (2016) [8], Ladurner et al. (2005) [10], Amiri-Nikpour et al. (2014) [11], Chang et al. (2016) [12], Muresanu et al. (2016) [13], Khasanova and Kalinin (2023) [14], Homberg et al. (2025) [15], Skvortsova et al. (2004) [16], Heiss et al. (2012) [17], Shamalov et al. (2010) [19], Guekht et al. (2017) [20], Gharagozli et al. (2017) [21], and Stan et al. (2017) [22].

Inclusion and Exclusion Criteria

We included randomized, double- or single-blind, placebo-controlled clinical trials that evaluated the use of intravenous Cerebrolysin in adult patients (aged ≥18 years) with a diagnosis of acute ischemic stroke confirmed by imaging. Trials had to report at least one of the following outcomes: change in NIHSS, proportion of patients with mRS 0-2, SAEs, all-cause mortality, or HT. Studies were excluded if they involved non-randomized designs, mixed intervention arms with other neuroprotectants, unavailable outcome data, or duplicate reporting of previously published data sets. Full-text articles were required; abstracts, conference proceedings, and case reports were excluded, as these sources typically lack sufficient methodological detail to allow accurate risk-of-bias assessment or extraction of outcome data. No restriction was applied to the follow-up duration, sample size, or treatment dose and intervention initiation time. All records were screened independently by two reviewers using Rayyan software (Rayyan Systems Inc., Cambridge, MA, US). Discrepancies in eligibility were resolved by discussion. Full texts of potentially eligible articles were retrieved and screened in detail. For non-English publications, English abstracts were reviewed first, and full texts were translated if the inclusion criteria appeared to be met. Study-level data were extracted using a pre-piloted Excel extraction form that included trial design, population characteristics, sample sizes, baseline NIHSS, intervention dose and timing, follow-up duration, and outcome results. Missing or unclear data were clarified through contact with the original study authors when possible. In the event of numerical inconsistencies, estimates were back-calculated from available summary statistics or extracted digitally from figures using WebPlotDigitizer.

Risk of Bias and Statistical Analysis

All included studies were assessed using the RoB 2.0 tool, evaluating domains including randomization, deviations from intended interventions, missing outcome data, outcome measurement, and selective reporting. Each trial was independently rated by two reviewers, with disagreements resolved by consensus. Results were stratified by outcome type and follow-up day when possible. Efficacy outcomes included NIHSS change (mean difference (MD)) and mRS 0-2 (risk ratio (RR)), while safety outcomes included SAEs, mortality, and HT. We used a random-effects model with DerSimonian-Laird estimators for all pooled analyses to account for clinical and statistical heterogeneity. For continuous outcomes, MDs and 95% confidence intervals (CIs) were calculated; for dichotomous outcomes, RRs were used. Between-study heterogeneity was quantified using the I² statistic. Sensitivity analyses were conducted by sequentially removing individual studies, and funnel plots were used to assess publication bias when at least five studies were available per outcome. All statistical analyses were performed using R software (version 4.3.2) (R Foundation for Statistical Computing, Vienna, Austria) with the “meta” and “metafor” packages. The certainty of evidence was evaluated (version 4.3.2) with the “meta” and “metafor” packages. The certainty of evidence was evaluated using the GRADE approach across all primary outcomes.

Results

**Figure 1:**
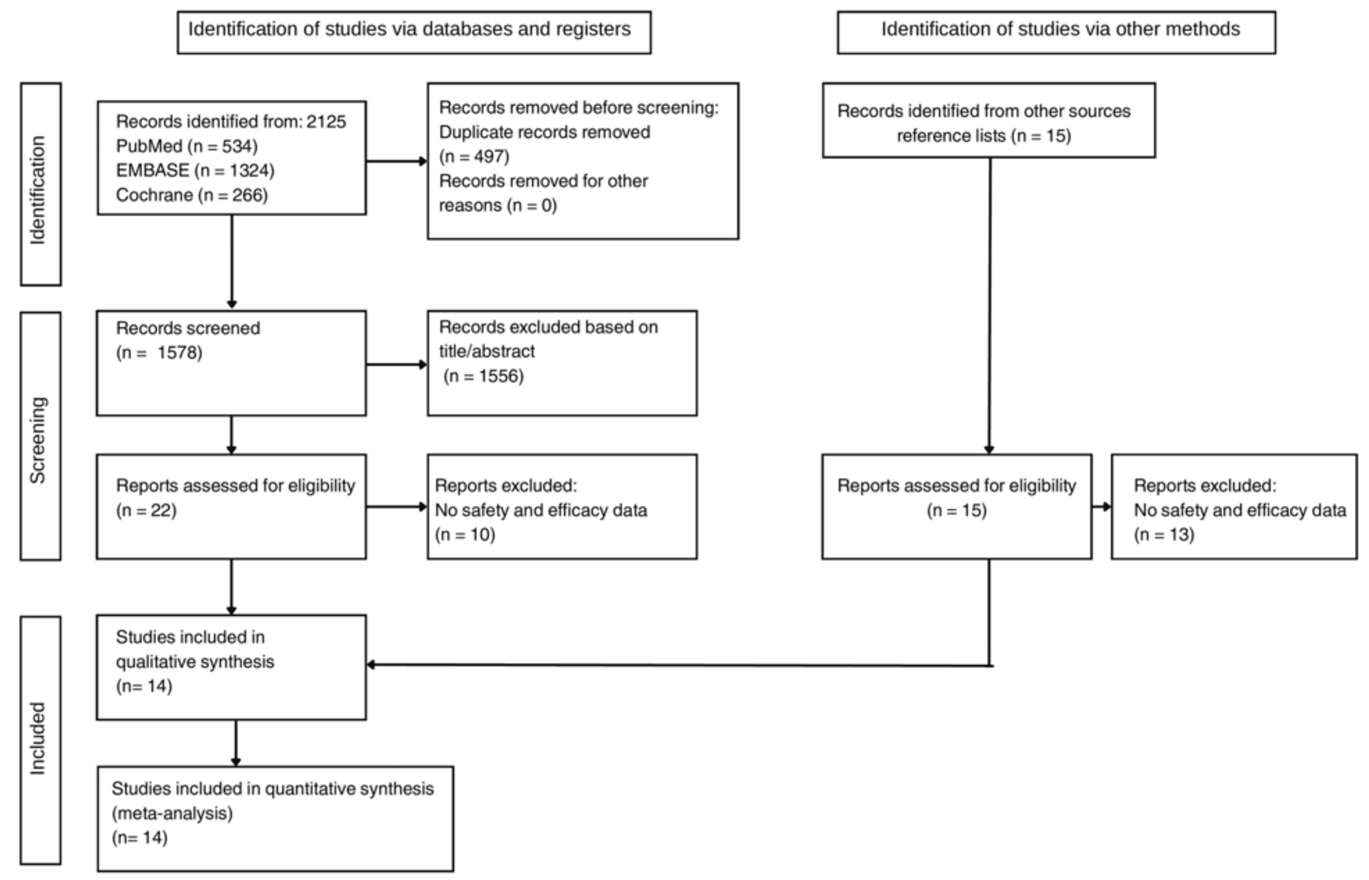
PRISMA 2020 flow diagram of study selection. Flowchart showing the number of records identified, screened, excluded, and ultimately included in the meta-analysis of Cerebrolysin for acute ischemic stroke. PRISMA: Preferred Reporting Items for Systematic reviews and Meta-Analyses

The systematic search identified 2,125 records through electronic databases-PubMed (n = 534), Embase (n = 1,324), and Cochrane Library (n = 266) and an additional 15 records from reference lists. After removing 497 duplicates, 1,578 records were screened by title and abstract. Of these, 1,556 were excluded, and 22 full-text articles were assessed for eligibility. A total of 14 RCTs met the inclusion criteria and were included in both the qualitative and quantitative syntheses (Figure 1).

**Figure 2:**
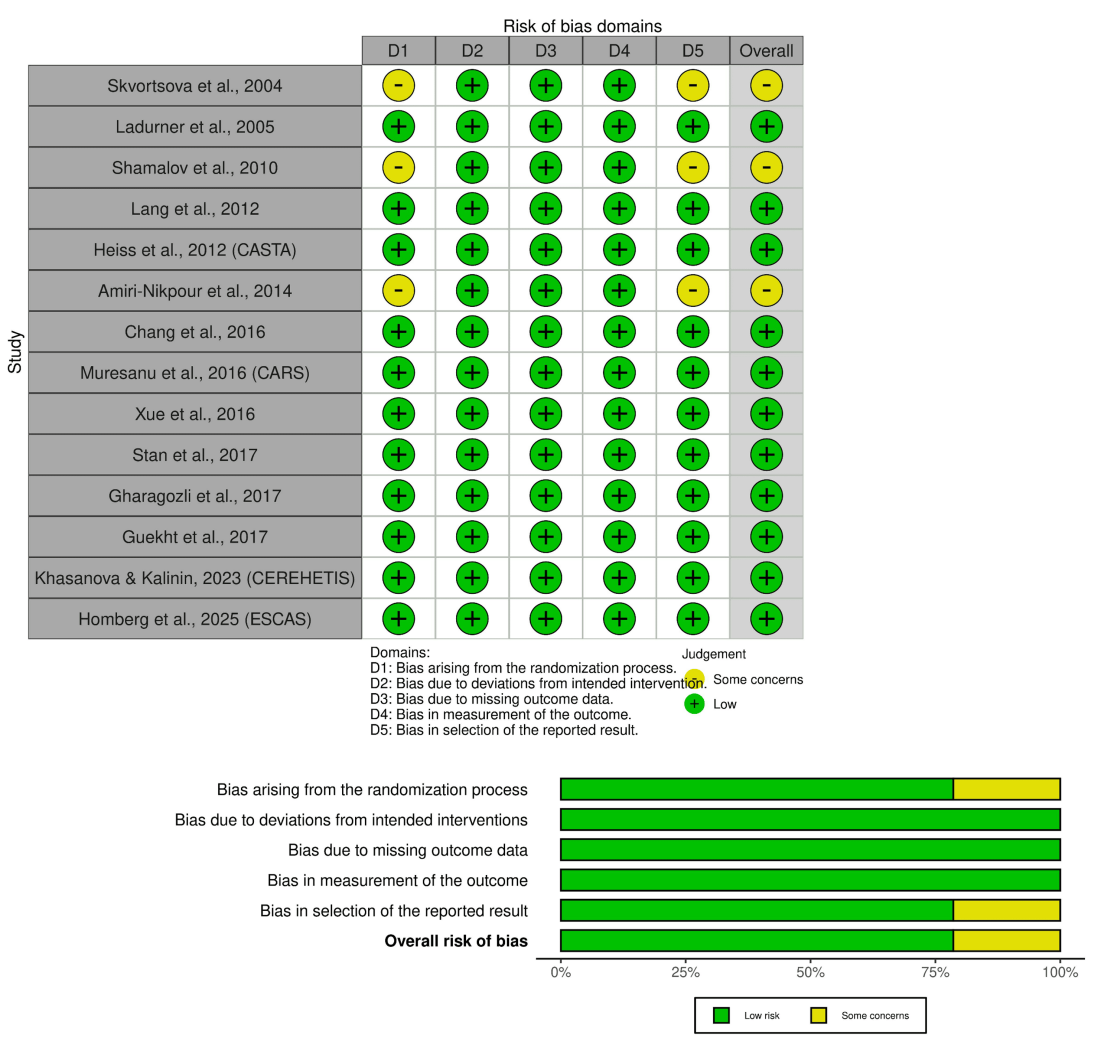
Risk of bias summary: traffic light plot and overall risk of bias across studies: bar plot. Domain-level risk of bias judgments for each included randomized controlled trial, assessed using the Cochrane RoB 2.0 tool. Studies included: Skvortsova et al. (2004) [16], Ladurner et al. (2005) [10], Shamalov et al. (2010) [19], Amiri-Nikpour et al. (2014) [11], Chang et al. (2016) [12], Stan et al. (2017) [22], Gharagozli et al. (2017) [21], Heiss et al. (2012, CASTA) [17], Muresanu et al. (2016, CARS) [13], Khasanova and Kalinin (2023, CEREHETIS) [14], Homberg et al. (2025, ESCAS) [15], Lang et al. (2012) [7], Guekht et al. (2017) [20], and Xue et al. (2016) [8]. Judgments were made across five domains of bias according to the RoB 2.0 tool. D1: randomization process; D2: deviations from intended interventions; D3: missing outcome data; D4: outcome measurement; D5: selective reporting

A total of 14 RCTs (N = 2,884) published between 2001 and 2023 were included. All studies employed parallel-group designs with placebo controls; 13 were double-blinded, and one used blinded outcome assessment. Patient populations were broadly balanced in age (~63-66 years), sex (~60%-65% male), and comorbidities (Table 1). Sample sizes ranged from 40 to 1,070. Most trials enrolled patients with moderate acute ischemic stroke (baseline NIHSS ~9-13). Cerebrolysin dosing was typically 30 mL/day (11 trials); three studies tested 50 mL or multiple doses. Treatment duration ranged from 10 to 21 days, initiated within 24 hours in nine trials, 24-72 hours in two, and up to seven days in one. Eight trials assessed outcomes at 90 days and others at 21-30 days (Table 2). Risk of bias assessment showed that 12 of the 14 included RCTs were judged to have low overall risk of bias. Two studies had some concerns, mainly related to unclear allocation concealment or lack of assessor blinding. No trial was rated as high risk (Figure 2). Additionally, three large trials were industry-sponsored; this was considered in the GRADE framework as a potential source of bias.

**Table 1:** Demographics of included studies. Baseline characteristics of patient populations across all included randomized controlled trials (RCTs), including age and sex distribution.

Study	Age (Cerebrolysin)	Age (placebo)	Male % (Cerebrolysin)	Male % (placebo)
Skvortsova et al. (2004) [16]	45–85	45–85	Not reported	Not reported
Ladurner et al. (2005) [10]	65; 1.17	65; 1.32	60.3%	55.9%
Shamalov et al. (2010) [19]	45–85	45–85	Not reported	Not reported
Heiss et al. (2012) [17]	65.0 ± 12.22	65.6 ± 11.71	59.6%	60.4%
Lang et al. (2012) [7]	65.6 ± 11.30	67.0 ± 10.56	66.7%	62.7%
Amiri-Nikpour et al. (2014) [11]	60 ± 9.6	60.1 ± 10	51.2%	47.6%
Guekht et al. (2017) [20]	63.8	63.8	59.7%	59.7%
Muresanu et al. (2016) [13]	64.9 ± 9.8	63.0 ± 10.6	67.3%	60.6%
Chang et al. (2016) [12]	64.7 ± 10.1	63.0 ± 10.6	82.9%	72.7%
Xue et al. (2016) [8]	66.5 ± 8.1	68.4 ± 4.2	45%	50%
Gharagozli et al. (2017) [21]	69.0 ± 10.7	66.5 ± 12.2	54%	52%
Stan et al. (2017) [22]	62.96 ± 10.9	65.23 ± 11.1	63.3%	66.5%
Khasanova and Kalinin (2023) [14]	63.5 (56–71)	68 (60–77)	60.3%	54.9%
Homberg et al. (2025) [15]	70.5 ± 11.2	67.7 ± 11.0	56.1%	53%

**Table 2:** Clinical and treatment characteristics of included studies. Study design elements for each RCT, including sample size, country, endpoints, follow-up day, treatment dose and duration, time to treatment initiation, comparator, and cotherapies. RCT: randomized controlled trial; C: Cerebrolysin; P: placebo; NIHSS: National Institutes of Health Stroke Scale; mRS: modified Rankin score

Author (year)	Sample size (C/P)	Countries	Endpoint	Follow-up day	Initiation window	Cerebrolysin regimen	Comparator	Cotherapies
Skvortsova et al. (2004) [16]	20/20	Russia, Romania	MRI infarct volume at day 30	30	Within 12 h	10 or 50 mL/day for 10 days	Placebo (0.9% saline)	+ ASA + pentoxifylline
Ladurner et al. (2005) [10]	78/68	Austria, Czech Republic, Hungary	CNS at day 21	21	Within 24 h	50 mL/day for 121 days	Placebo (0.9% saline)	None
Shamalov et al. (2010) [19]	24/23	Russia	MRI infarct volume at day 30	30	Within 12 h	50 mL/day for 10 days	Placebo (0.9% saline)	+ ASA 100 mg/day x 10 days
Heiss et al. (2012) [17]	529/541	China, Hong Kong, South Korea, Myanmar	Composite of NIHSS, mRS, BI at day 90	90	Within 12 h	30 mL/day for 10 days	Placebo (0.9% saline)	+ ASA 100 mg/day x 90 days
Lang et al. (2012) [7]	60/59	Austria, Croatia, Czech Republic, Slovakia, Slovenia	mRS at day 90	90	Within 3 h	30 mL/day for 10 days	Placebo (0.9% saline)	+ rt-PA
Amiri-Nikpour et al. (2014) [11]	23/23	Iran	NIHSS at day 30, 60, 90	90	6–24 h	30 mL/day for 10 days	Placebo	+ ASA
Guekht et al. (2017) [20]	224/224	Russia	ARAT at day 90	90	24–72 h	30 mL/day for 21 days	Placebo	+ basic therapy
Muresanu et al. (2016) [13]	104/104	Romania, Ukraine, Poland	ARAT at day 90	90	24–72 h	30 mL/day for 21 days	Placebo	+ basic therapy
Chang et al. (2016) [12]	35/35	Korea	FMA-T at day 29	29	Within 7 days	30 mL/day for 21 days	Placebo (0.9% saline)	None
Xue et al. (2016) [8]	28/29	China	NIHSS and BI day 30	30	Within 12 h	30 mL/day for 10 days	NBP	+ basic therapy
Gharagozli et al. (2017) [21]	50/50	Iran	NIHSS at day 30	30	Within 18 h	30 mL/day (1–7), 10 mL/day (weeks 2–4)	Placebo (0.9% saline)	+ basic therapy
Stan et al. (2017) [22]	30/30	Romania	NIHSS at day 30	30	Within 48 h	30 mL/day for 10 days	Placebo	None
Khasanova and Kalinin (2023) [14]	126/215	Russia	NIHSS at day 14	14	Within 24 h	30 mL/day for 10 days	Placebo	Not reported
Homberg et al. (2025) [15]	66/66	Germany	NIHSS, mRS, AQ	30	Within 7 days	30 mL/day for 21 days	Placebo	+ speech therapy

Neurological Improvement (ΔNIHSS)

**Figure 3:**
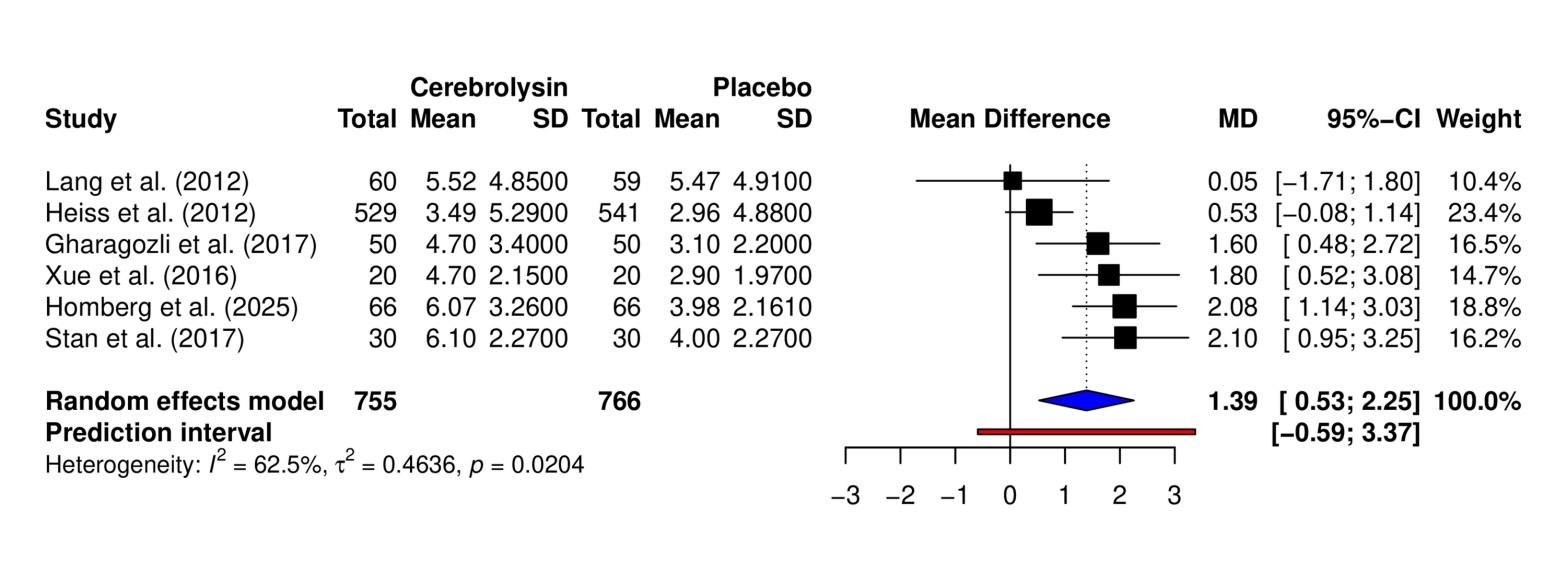
Forest plot of NIHSS score change (ΔNIHSS) comparing Cerebrolysin versus placebo. Pooled analysis of six randomized controlled trials assessing change in NIHSS score from baseline to follow-up. Effect estimates are presented as mean differences (MDs) with 95% confidence intervals (CIs), calculated using the random-effects model. Studies included: Lang et al. (2012) [7], Heiss et al. (2012) [17], Gharagozli et al. (2017) [21], Xue et al. (2016) [8], Homberg et al. (2025) [15], and Stan et al. (2017) [22]. NIHSS: National Institutes of Health Stroke Scale; SD: standard deviation

**Figure 4:**
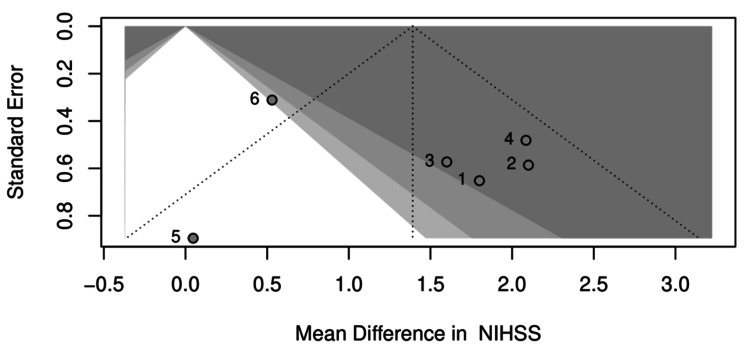
Funnel plot assessing publication bias in the NIHSS outcome. Visual assessment of potential publication bias across studies reporting NIHSS change scores. NIHSS: National Institutes of Health Stroke Scale

**Figure 5:**
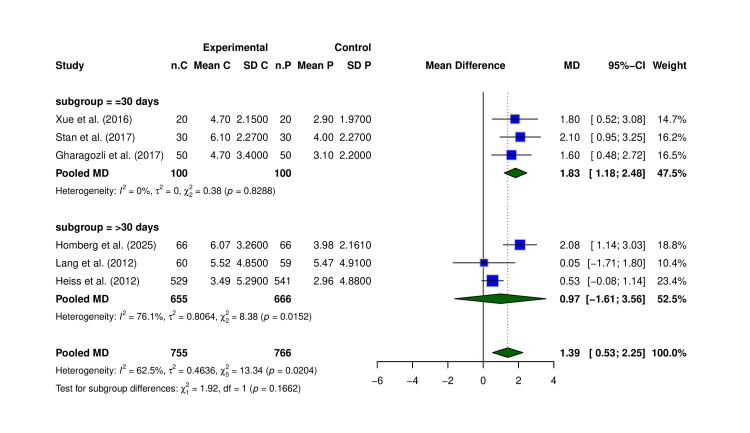
Subgroup analysis of NIHSS outcome by follow-up duration. Comparison of effect size stratified by follow-up day (≤30 vs. >30). Random-effects model (M-H) used. Studies included in the ≤30 days subgroup: Xue et al. (2016) [8], Stan et al. (2017) [22], and Gharagozli et al. (2017) [21]. Studies included in the >30 days subgroup: Homberg et al. (2025) [15], Lang et al. (2012) [7], and Heiss et al. (2012) [17]. NIHSS: National Institutes of Health Stroke Scale; CI: confidence interval; MD: mean difference; SD: standard deviation; C: Cerebrolysin; P: placebo

Nine of 14 trials showed numerically greater NIHSS improvement with Cerebrolysin (Table 3). Only six studies (N = 1,521) that reported the mean change in NIHSS were included in the meta-analysis. Pooled analysis showed a significant benefit: MD +1.39 points (95% CI: 0.53-2.25, p = 0.0204; I² = 62.5%) (Figure 3). Although modest in absolute value, even a one-point shift on the NIHSS can be clinically meaningful, as it may represent improved motor strength, speech, or independence in daily activities, potentially altering functional outcomes in real-world recovery. The effect remained robust across sensitivity analyses and was not driven by any single study. The funnel plot showed minor asymmetry but no clear publication bias (Figure 4). Subgroup analysis is performed between follow-up days ≤ 30 and >30, though not statistically different (Figure 5).

**Table 3:** NIHSS outcome summary from included studies. Baseline and follow-up NIHSS scores in Cerebrolysin (C) and placebo (P) groups across included trials. Data include sample sizes, mean change in NIHSS, standard deviations, and follow-up time points. NIHSS: National Institutes of Health Stroke Scale

Author (year)	Sample size (C)	Sample size (P)	Baseline NIHSS (C)	Baseline NIHSS (P)	Final NIHSS (C)	Final NIHSS (P)	SD/IQR C (final)	SD/IQR P (final)	Follow-up day
Skvortsova et al., 2004 [16]	20	20	13.1	12.6	Not reported	Not reported	Not reported	Not reported	30
Ladurner et al., 2005 [10]	78	68	Not reported (CNS used)	Not reported (CNS used)	Not reported	Not reported	≈0.79 (CNS-derived)	≈1.15 (CNS-derived)	90
Shamalov et al., 2010 [19]	24	23	7.7	8.6	Not reported	Not reported	Not reported	Not reported	30
Lang et al., 2012 [7]	60	59	12.3 ± 5.39	11.0 ± 5.44	6.78	5.53	±1.39	±1.35	90
Heiss et al., 2012 [17]	529	541	8.7 ± 4.83	8.5 ± 4.80	5.21	4.34	≈5.29 (from SE)	≈4.88 (from SE)	90
Amiri-Nikpour et al., 2014 [11]	23	23	Median 14 (IQR 13–15)	Median 14 (IQR 12–16)	Median 9	Median 11	IQR 8–10	IQR 10–13.5	90
Chang et al., 2016 [12]	34	32	8.4 ± 5.8	7.0 ± 4.9	Not reported	Not reported	Not reported	Not reported	90
Muresanu et al., 2016 [13]	104	104	9.1 ± 3.2	9.2 ± 3.2	Not reported	Not reported	Not reported	Not reported	90
Xue et al., 2016 [8]	20	20	10.60 ± 4.74	10.20 ± 3.72	5.90	7.30	±3.96	±4.78	21
Stan et al., 2017 [22]	30	30	8.9 ± 3.42	7.8 ± 2.36	2.8	3.8	±2.27	±2.27	30
Gharagozli et al., 2017 [21]	50	50	11.1 ± 5.0	9.1 ± 4.8	6.2	6.0	±5.1	±4.8	30
Guekht et al., 2017 [20]	119	121	Not reported (combined: 6.8)	Not reported (combined: 6.8)	Not reported	Not reported	Not reported	Not reported	21
Khasanova and Kalinin, 2023 [14]	126	215	Median 10 (IQR 6–14)	Median 10 (IQR 7–14)	Median 2	Median 3	IQR 1–6	IQR 2–7	14
Homberg et al., 2025 [15]	66	66	9.2 ± 4.5	8.6 ± 4.4	3.13	4.62	±3.26	±2.16	90

Functional Independence (mRS 0-2)

**Figure 6:**
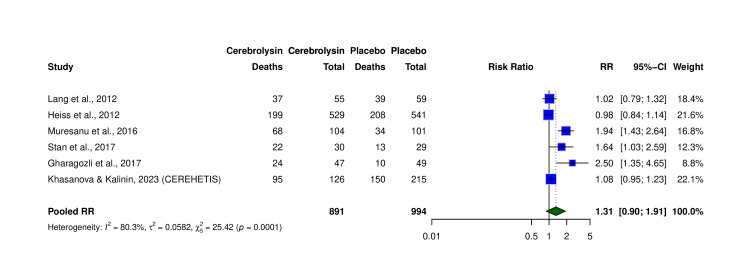
Forest plot of functional independence (mRS 0–2) at follow-up. Pooled analysis of six RCTs comparing the proportion of patients achieving mRS 0–2 in Cerebrolysin versus placebo groups. Risk ratios (RRs) with 95% CI calculated using the Mantel–Haenszel method, random-effects model. Studies included: Lang et al. (2012) [7], Heiss et al. (2012) [17], Muresanu et al. (2016) [13], Stan et al. (2017) [22], Gharagozli et al. (2017) [21], and Khasanova and Kalinin (2023, CEREHETIS) [14]. CI: confidence interval; mRS: modified Rankin score; RCT: randomized controlled trial

Six RCTs (N = 1,885) reported mRS 0-2 (Table 4). Pooled RR was 1.31 (95% CI: 0.90-1.91, p > 0.05; I² = 80.3%) (Figure 6). While not statistically significant, absolute rates of independence were ~49.9% with Cerebrolysin versus ~45.7% with placebo, corresponding to a possible ~4.2% higher rate of functional independence. The GRADE certainty was moderate, downgraded for imprecision. Funnel test and formal bias testing were not performed due to the study count.

**Table 4:** mRS outcome summary from included studies. Proportions of patients achieving functional independence (mRS 0–2) at follow-up. Data include mRS response rates and follow-up days. C: Cerebrolysin; P: placebo; mRS: modified Rankin score

Author (year)	Sample size (C)	Sample size (P)	mRS 0–2 (C)	mRS 0–2 (P)	Follow-up day
Skvortsova et al., 2004 [16]	20	20	Not reported	Not reported	30
Ladurner et al., 2005 [10]	78	68	Not reported	Not reported	90
Shamalov et al., 2010 [19]	24	23	Not reported	Not reported	30
Lang et al., 2012 [7]	55	59	37	39	90
Heiss et al., 2012 [17]	529	541	≈199 (37.6%)	≈208 (38.5%)	90
Amiri-Nikpour et al., 2014 [11]	23	23	Not reported	Not reported	90
Chang et al., 2016 [12]	34	32	Not reported	Not reported	90
Muresanu et al., 2016 [13]	104	101	≈68 (65.4%)	≈34 (33.7%)	90
Xue et al., 2016 [8]	20	20	Not reported	Not reported	21
Stan et al., 2017 [22]	30	29	22	13	30
Gharagozli et al., 2017 [21]	47	49	24	10	30
Guekht et al., 2017 [20]	≈119	≈121	Not reported	Not reported	21
Khasanova and Kalinin, 2023 [14]	126	215	95	150	90
Homberg et al., 2025 [15]	66	66	Not reported	Not reported	90

Serious Adverse Events

**Figure 7:**
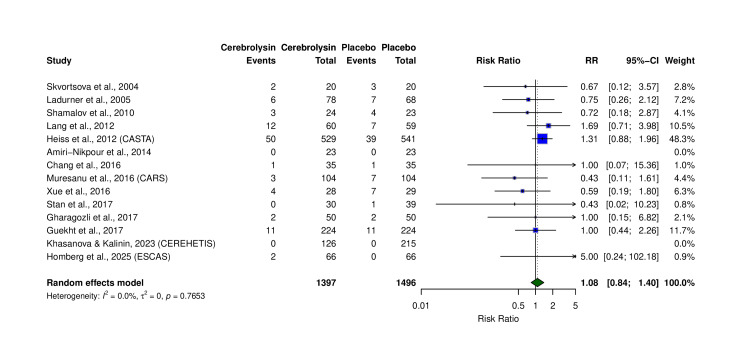
Forest plot of serious adverse events (SAEs) across included trials. Comparison of patients in the Cerebrolysin and placebo groups. Analysis used the Mantel–Haenszel method, random-effects model. Studies included: Skvortsova et al. (2004) [16], Ladurner et al. (2005) [10], Shamalov et al. (2010) [19], Lang et al. (2012) [7], Heiss et al. (2012, CASTA) [17], Amiri-Nikpour et al. (2014) [11], Chang et al. (2016) [12], Muresanu et al. (2016, CARS) [13], Stan et al. (2017) [22], Gharagozli et al. (2017) [21], Guekht et al. (2017) [20], Khasanova and Kalinin (2023, CEREHETIS) [14], and Homberg et al. (2025, ESCAS) [15]. RR: risk ratio; CI: confidence interval

Fourteen trials (N = 2,893) reported SAEs (Table 5). There was no statistically significant difference in the risk of SAEs between the Cerebrolysin and placebo groups (RR = 1.08, 95% CI: 0.84-1.40, p = 0.76; I² = 0.0%) (Figure 7). SAE rates were 6.87% in Cerebrolysin vs. 5.95% in placebo. No excess adverse events were detected with Cerebrolysin, though larger trials are required to confirm safety for rare complications. Results were consistent with prior safety analyses.

**Table 5:** Serious adverse events (SAE) reported across included studies. Number of SAEs reported in the Cerebrolysin (C) and placebo (P) groups. Data include event counts and sample sizes.

Author (year)	Sample size (C)	Sample size (P)	SAE count (C)	SAE count (P)	Follow-up day(s)
Skvortsova et al., 2004 [16]	20	20	2	3	90 days
Ladurner et al., 2005 [10]	78	68	6	7	90 days
Shamalov et al., 2010 [19]	24	23	3	4	90 days
Lang et al., 2012 [7]	60	59	12	7	90 days
Heiss et al., 2012 [17]	529	541	50	39	90 days
Amiri-Nikpour et al., 2014 [11]	23	23	0	0	90 days
Chang et al., 2016 [12]	35	35	1	1	90 days
Muresanu et al., 2016 [13]	104	104	3	7	90 days
Xue et al., 2016 [8]	28	29	4	7	21 days
Stan et al., 2017 [22]	30	39	0	1	30 days
Gharagozli et al., 2017 [21]	50	50	2	2	30 days
Guekht et al., 2017 [20]	224	224	11	11	90 days
Khasanova and Kalinin, 2023 [14]	126	215	0	0	90 days
Homberg et al., 2025 [15]	66	66	2	0	90 days

Mortality

**Figure 8:**
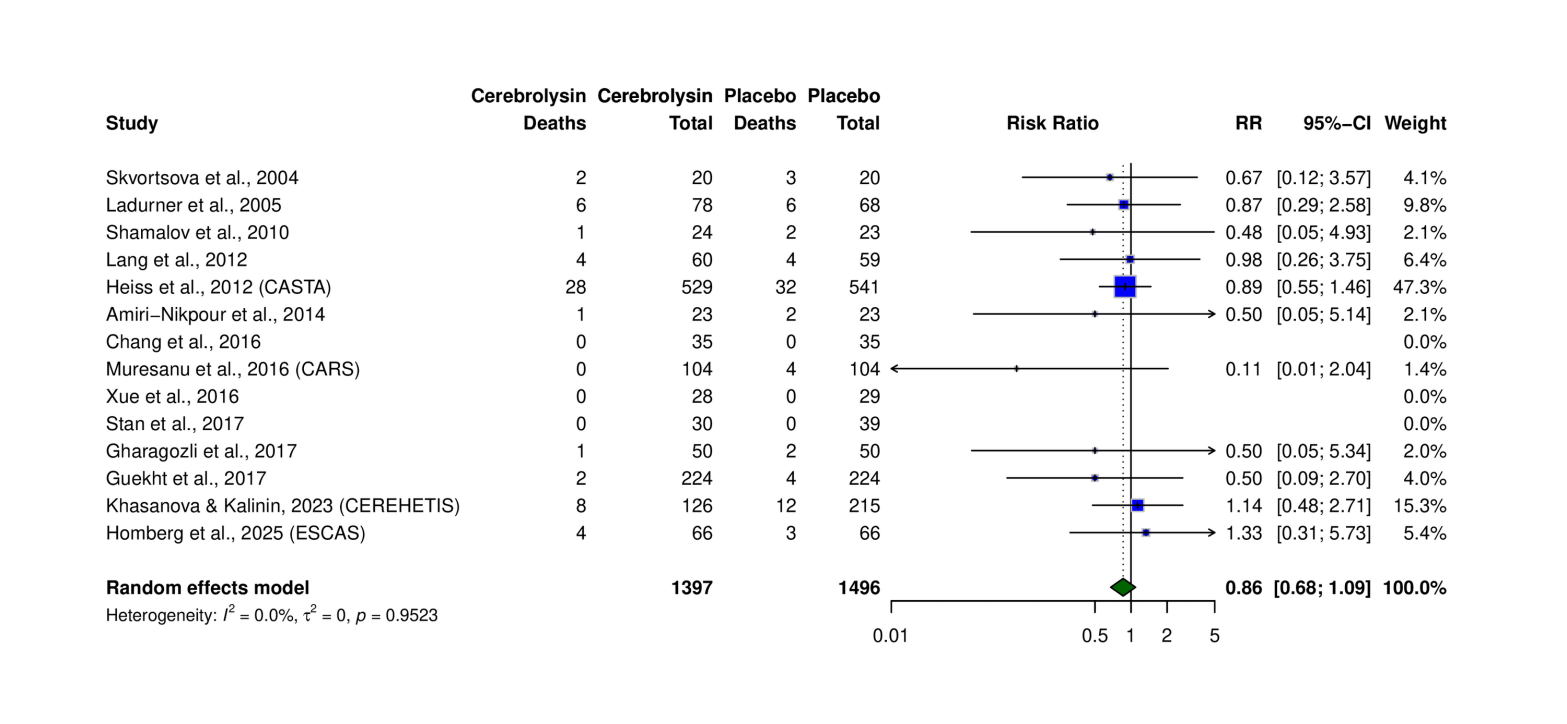
Forest plot of all-cause mortality in Cerebrolysin versus placebo from 14 trials. Calculated using M-H, random-effects model. Studies included: Skvortsova et al. (2004) [16], Ladurner et al. (2005) [10], Shamalov et al. (2010) [19], Lang et al. (2012) [7], Heiss et al. (2012, CASTA) [17], Amiri-Nikpour et al. (2014) [11], Chang et al. (2016) [12], Muresanu et al. (2016, CARS) [13], Xue et al. (2016) [8], Stan et al. (2017) [22], Gharagozli et al. (2017) [21], Guekht et al. (2017) [20], Khasanova and Kalinin (2023, CEREHETIS) [14], and Homberg et al. (2025, ESCAS) [15]. RR: risk ratio; CI: confidence interval

All-cause mortality from 14 trials (N = 2,893) showed no significant difference: RR = 0.86 (95% CI: 0.68-1.09, p = 0.95; I² = 0.0%) (Table 6, Figure 8). Absolute death rates were 4.08% (Cerebrolysin) vs. 4.95% (placebo). Although not statistically significant, directionality consistently favored Cerebrolysin across trials.

**Table 6:** Mortality events reported in each study arm. Data include sample sizes and the number of death event rates. C: Cerebrolysin; P: placebo

Author (year)	Sample size (C)	Sample size (P)	Deaths (C)	Deaths (P)	Follow-up day
Skvortsova et al., 2004 [16]	20	20	2	3	90
Ladurner et al., 2005 [10]	78	68	6	6	90
Shamalov et al., 2010 [19]	24	23	1	2	90
Lang et al., 2012 [7]	60	59	4	4	30
Heiss et al., 2012 [17]	529	541	28	32	90
Amiri-Nikpour et al., 2014 [11]	23	23	1	2	90
Chang et al., 2016 [12]	35	35	0	0	90
Muresanu et al., 2016 [13]	104	104	0	4	90
Xue et al., 2016 [8]	28	29	0	0	90
Stan et al., 2017 [22]	30	39	0	0	90
Gharagozli et al., 2017 [21]	50	50	1	2	21
Guekht et al., 2017 [20]	224	224	2	4	30
Khasanova and Kalinin, 2023 [14]	126	215	8	12	90
Homberg et al., 2025 [15]	66	66	4	3	90

Hemorrhagic Transformation

**Figure 9:**
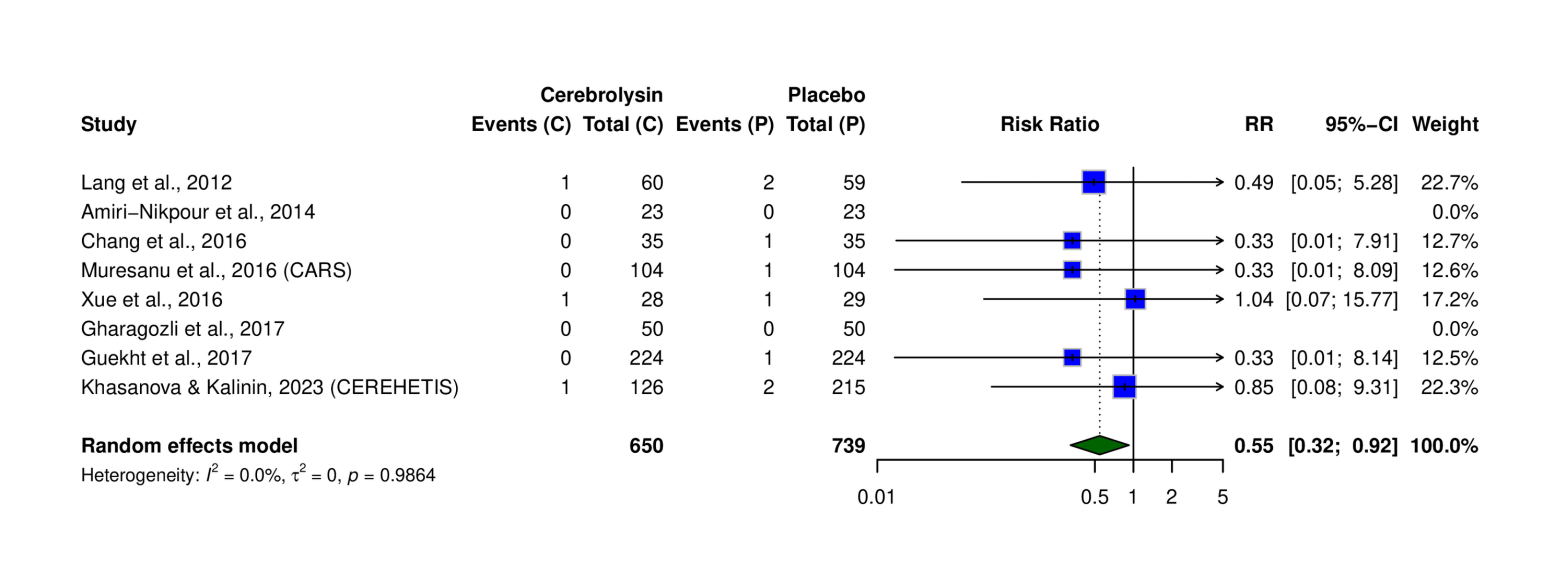
Forest plot of hemorrhagic transformation events. Analysis of eight RCTs comparing the incidence of hemorrhagic transformation in the Cerebrolysin (C) and placebo (P) groups. Random-effects model (M-H) used. Studies included: Lang et al. (2012) [7], Amiri-Nikpour et al. (2014) [11], Chang et al. (2016) [12], Muresanu et al. (2016, CARS) [13], Gharagozli et al. (2017) [21], Guekht et al. (2017) [20], Khasanova and Kalinin (2023, CEREHETIS) [14], and Xue et al. (2016) [8]. RR: risk ratio; CI: confidence interval; RCT: randomized controlled trial

Eight trials (N = 1,389) assessed the incidence of HT (Table 7). Cerebrolysin significantly reduced the risk of HT compared to placebo (RR = 0.55, 95% CI: 0.32-0.92). No heterogeneity was observed among studies (I² = 0.0%, τ² = 0, p = 0.9864), indicating a consistent protective effect across trials (Figure 9). The certainty of evidence across all outcomes was assessed using the GRADE approach. The primary outcome (ΔNIHSS) was rated as moderate certainty due to risk of bias in some studies. Functional independence (mRS 0-2) was rated as low certainty, downgraded for inconsistency and imprecision. Safety outcomes, including SAEs and mortality, were rated as moderate certainty, while HT was rated as high certainty. Full GRADE ratings are summarized in Table 8.

**Table 7:** Hemorrhagic transformation (HT) events reported across included studies. Data include event counts in the Cerebrolysin (C) and placebo (P) groups and the total sample size.

Author (year)	HT events (C)	Sample size (C)	HT events (P)	Sample size (P)	Follow-up day
Lang et al., 2012 [7]	1	60	2	59	Day 90
Amiri-Nikpour et al., 2014 [11]	0	23	0	23	Day 90
Chang et al., 2016 [12]	0	35	1	35	Day 90
Muresanu et al., 2016 (CARS) [13]	0	104	1	104	Day 90
Xue et al., 2016 [8]	1	28	1	29	Day 90
Gharagozli et al., 2017 [21]	0	50	0	50	Day 90
Guekht et al., 2017 [20]	0	224	1	224	Day 90
Khasanova and Kalinin, 2023 (CEREHETIS) [14]	1	126	2	215	Day 90

**Table 8:** Summary of GRADE assessment for primary and secondary outcomes. Includes outcome description, number of studies, overall certainty level, and rationale for any downgrading. GRADE: Grading of Recommendations, Assessment, Development, and Evaluation; RCT: randomized controlled trial; mRS: modified Rankin score; RR: risk ratio; MD: mean difference; CI: confidence interval

Outcome	No. of participants (studies)	Follow-up	Risk of bias	Inconsistency	Indirectness	Imprecision	Publication bias	Certainty	Effect summary (95% CI)	Comments
NIHSS (Δ mean score)	1,521 (6 RCTs)	30–90 days	All low risk	Serious (I² = 62.5%)	Not serious	Not serious	Possible (funnel plot asymmetry)	Moderate	MD: 1.39 (0.53–2.25)	Consistent benefit but heterogeneity present
mRS 0–2 (functional independence)	1,885 (6 RCTs)	30–90 days	All low risk	Serious (I² = 80.3%)	Not serious	Not serious	None detected	Moderate	RR: 1.31 (0.90–1.91)	Benefit possible but with heterogeneity present
Serious adverse events (SAEs)	2,893 (14 RCTs)	30–90 days	2/14 with some concerns	Not serious (I² = 0%)	Not serious	Not serious	None detected	High	RR: 1.08 (0.84–1.40)	No increase in SAE risk
Mortality	2,893 (14 RCTs)	30–90 days	2/14 with some concerns	Not serious (I² = 0%)	Not serious	Not serious	None detected	High	RR: 0.86 (0.68–1.09)	Trend toward reduced mortality
Hemorrhagic transformation (HT)	1,389 (8 RCTs)	30–90 days	1/8 with some concerns	Not serious (I² = 0%)	Not serious	Not serious	None detected	High	RR: 0.55 (0.32–0.92)	Statistically significant reduction in HT

Discussion

This updated meta-analysis of 14 RCTs involving 2,884 patients provides the most comprehensive evaluation to date of Cerebrolysin’s role in early recovery after acute ischemic stroke. The included trials primarily enrolled adults with moderate baseline stroke severity (NIHSS ~9-13), tested intravenous Cerebrolysin at doses of 30-50 mL/day for 10-21 days, and assessed outcomes at 21-90 days of follow-up. Thus, the findings are most applicable to patients with moderate-to-severe ischemic stroke treated within the first week of onset.

Compared to placebo, Cerebrolysin was associated with significantly greater neurological improvement (MD: +1.39 NIHSS points), suggesting a modest but clinically relevant benefit when added to standard stroke care. Although small in absolute value, this modest improvement may, in some cases, contribute to clinically meaningful threshold shifts (e.g., from needing assistance to regaining independence), though further trials are required to confirm this hypothesis, which has been suggested but not conclusively demonstrated in prior clinical studies [16,17]. While the pooled effect on functional independence (mRS 0-2) was not statistically significant, the pooled estimate suggested a possible higher rate of independence (~4.2% absolute difference). Limited sample size, heterogeneity, and differences in follow-up duration (30 vs. 90 days) may account for the lack of statistical significance, but the pattern remains encouraging, particularly in patients with moderate-to-severe stroke.

Importantly, our analysis confirmed Cerebrolysin’s favorable safety profile. No excess adverse events were detected, though larger trials are required to confirm safety for rare complications. There were no significant increases in SAEs, mortality, or HT. All point estimates slightly favored Cerebrolysin, aligning with prior findings from Strilciuc et al. and Bornstein et al., which concluded the drug was as safe as placebo in acute stroke, consistent with previous clinical trial results [5,6]. Furthermore, Cerebrolysin did not increase HT risk even when used alongside reperfusion therapies, consistent with both clinical trials and mechanistic data suggesting stabilization of the blood-brain barrier [7,12].

Our findings also address key limitations of previous reviews. For example, the 2020 Cochrane meta-analysis and earlier trials, including CASTA, reported mixed results, in part due to the inclusion of mild strokes and heterogeneous outcome measures [17,18]. In contrast, our analysis focused on studies using comparable endpoints and included recently published trials (e.g., ESCAS and CEREHETIS) not captured in previous reviews, thereby offering a more up-to-date and nuanced synthesis [14,15]. Additionally, we applied rigorous RoB 2.0 and GRADE frameworks, enhancing the transparency and reliability of the evidence. Some heterogeneity remains, particularly regarding follow-up duration, patient severity, and treatment timing. Exploratory data hint at greater efficacy in more severe strokes-consistent with the hypothesis that neurorecovery agents may be most beneficial in patients with substantial initial deficits [18].

Publication bias was formally assessed using funnel plots for NIHSS, which showed minor asymmetry but no clear publication bias. The GRADE framework rated the certainty of evidence as moderate for NIHSS, moderate for mRS 0-2, and high for safety outcomes (SAEs, mortality, or HT). A potential limitation of our review is the exclusion of conference abstracts and proceedings. While this approach minimizes risk of bias due to incomplete reporting, it may also have led to omission of relevant recent or unpublished evidence, including potentially negative trials.

Overall, Cerebrolysin appears safe and potentially beneficial for improving early recovery after stroke. Future large-scale RCTs targeting functionally relevant endpoints in specific subgroups (e.g., moderate-to-severe stroke and post-recanalization) are warranted to confirm these findings and optimize dosing strategies.

## Conclusions

This updated meta-analysis of 14 RCTs provides moderate-certainty evidence that Cerebrolysin improves early neurological recovery after acute ischemic stroke, with a statistically significant effect on NIHSS score change. Although the improvement in functional independence (mRS 0-2) did not reach statistical significance, the consistent trend across trials suggests a potential clinical benefit. Importantly, Cerebrolysin was not associated with increased risk of SAEs, mortality, or HT, reaffirming its favorable safety profile. Compared to earlier meta-analyses, our study incorporates recent trials and applies rigorous methodology, including RoB 2.0 and GRADE frameworks. Taken together, these findings indicate that Cerebrolysin appears safe and may offer benefits for early neurological recovery; however, evidence for functional independence remains inconclusive, warranting larger, well-powered trials to confirm these results and to further define optimal dosing and patient selection strategies.
